# Collapsing glomerulopathy in renal allograft biopsies: A study of nine cases

**DOI:** 10.4103/0971-4065.75220

**Published:** 2011

**Authors:** R. Gupta, A. Sharma, S. K. Agarwal, A. K. Dinda

**Affiliations:** Department of Pathology, All India Institute of Medical Sciences, New Delhi, India; 1 Department of Nephrology, All India Institute of Medical Sciences, New Delhi, India

**Keywords:** Collapsing glomerulopathy, histopathology, outcome, renal allograft

## Abstract

Collapsing glomerulopathy (CG) is considered to be a distinct clinicopathologic pattern of proliferative podocyte injury. The clinical significance of CG in renal allograft biopsies is yet not clear due to the scant data on the occurrence of CG in renal transplant recipients. We identified nine cases of CG in allograft biopsies over a period of 2 years. Detailed clinical information, including follow-up data, was collected and histopathological analysis performed. All the nine patients were males with a mean age at diagnosis of 32.4±11.2 years. The median posttransplantation duration at diagnosis of CG as 52 (range 12–98) months. All the patients presented with severe proteinuria and graft dysfunction. Histological analysis showed a median number of 8 glomeruli. The collapse of the glomerular tuft with visceral epithelial cell hyperplasia involved median of 2 glomeruli (range 1–4). At the last follow-up (mean duration 6 months), four patients had graft failure (return to dialysis) while four had functioning grafts. One patient was lost to follow-up. This series emphasizes the importance of this rare glomerular pathology as an important cause of graft dysfunction that may lead to allograft failure.

## Introduction

Collapsing glomerulopathy (CG) was recognized in 1978 as a variant of focal and segmental glomerulosclerosis (FSGS), especially associated with HIV infection.[1] Later reports showed the occurrence of CG in HIV-negative patients as well and this entity was called “collapsing FSGS.”[2] Recent studies suggest that CG is a distinct clinicopathologic entity, not related to FSGS due to the differences in the clinical presentation, histologic appearance, and outcome.[3]

The occurrence of CG in renal allografts has been reported as case reports and small studies.[4–7] In the previous studies, patients had presented with graft dysfunction and proteinuria, varying from few days in recurrent cases to many years after transplantation in *de novo* CG. The long-term outcome of the previously reported patients has been unfavorable with most of the patients returning to dialysis.[7,8] However, the appropriate therapeutic management of this rare pathology is still unclear. Only an occasional case report of CG in renal allografts from this subcontinent was found in the English literature.[4,9]

This series presents the clinical and pathologic features of nine renal transplant recipients with CG in allograft biopsies over a period of 2 years.

## Materials and Methods

All renal allograft biopsies received by our department between 2008 and 2009 were reviewed. CG was diagnosed on the basis of diffuse or focal, segmental or global glomerular capillary collapse with hyperplasia/hypertrophy of overlying visceral epithelial cells (podocytes). During the study period, nine such biopsies were identified.

Clinical data were obtained from a review of the patients’ medical records. The details recorded were age, sex, duration of transplant, pretransplant biopsy diagnosis, immunosuppressive regimen along with severity of proteinuria, and allograft dysfunction. Serological investigations for viral infections (HIV, hepatitis B [HBV], parvovirus) were also recorded. Prior rejection episodes and drug toxicity (especially calcineurin inhibitors [CNIs]) were documented.

Renal biopsy tissues were processed for light microscopy and immunofluorescence microscopy by standard techniques. For light microscopy, sections were stained with hematoxylin and eosin, periodic acid Schiff, and silver methenamine stains. Interstitial fibrosis and tubular atrophy were graded semiquantitatively on a scale of 0 to 3 (absent, up to 25%, 26–50%, >50%, respectively). In addition, cellular rejection was identified and graded according to the Banff 2007 update.[10] CNI toxicity was identified by the presence of peripheral nodular arteriolar hyalinosis and/or a “striped” pattern of interstitial fibrosis and tubular atrophy.

## Results

A total of 254 renal allograft biopsies were performed during the study period (2008–2009). Of these, nine showed features of CG, constituting 3.5% of all allograft biopsies. All these nine patients had undergone renal allograft biopsy for the evaluation of graft dysfunction and proteinuria.

All the nine patients were males with an age ranging from 19 to 58 years (average 32.44±11.2 years). These patients were diagnosed as CG in the allograft biopsy at a median of 52 months after transplantation (range 12-98 months). All the patients received allografts from living donors (mother 5, brother 2, sister 1, and wife 1). The native kidney disease was unknown in these patients. Of the nine patients, one presented with pedal edema while the other eight underwent biopsy for asymptomatic graft dysfunction (median serum creatinine 1.95 mg/dl; range 1.6–5.0 mg/dl). All the patients had proteinuria with 24-h urinary protein excretion ranging between 2.5 and 5.8 g/day. Three patients had hepatitis C viral infection prior to the renal transplant, but did not receive antiviral therapy for the same. All the nine patients were receiving steroid-based triple drug immunosuppression (cyclosporine A in six and tacrolimus in three). The serum CNI trough levels were within expected ranges in all the patients at the time of biopsy. The salient clinical features are summarized in Table 1.

**Table 1:** Clinical and laboratory parameters of nine patients with collapsing glomerulopathy

	Patient no.								
	1	2	3	4	5	6	7	8	9
Age (year)/sex	29/M	37/M	25/M	26/M	19/M	32/M	28/M	33/M	58/M
Time of biopsy (months)	63	32	96	52	19	75	12	22	90.5
Indication for biopsy	CGD	Pedal edema	CGD	CGD	CGD	CGD	CGD	CGD	CGD
S. Cr. (mg/dl)	3.4	1.6	1.8	2.2	5	1.7	1.95	1.6	4.9
Urine protein (g/24 h)	3.4	3.8	2.5	3.0	2.6	5.8	3.5	4.0	2.8
Hypertension	+	+	–	+	–	–	+	–	–
Follow-up (months)	2	6	12	6	NA	3	3	3	3
Outcome (S. Cr, mg/dl)	Graft failed	1.6	1.8	5.8	NA	5.8	2.1	1.4	5.2

S. Cr. = Serum creatinine; CGD = Chronic graft dysfunction; NA = Not available

None of the patients had a prior episode of acute rejection. All the patients were negative for HIV, HBV and parvovirus by serology.

**Figure 1:**
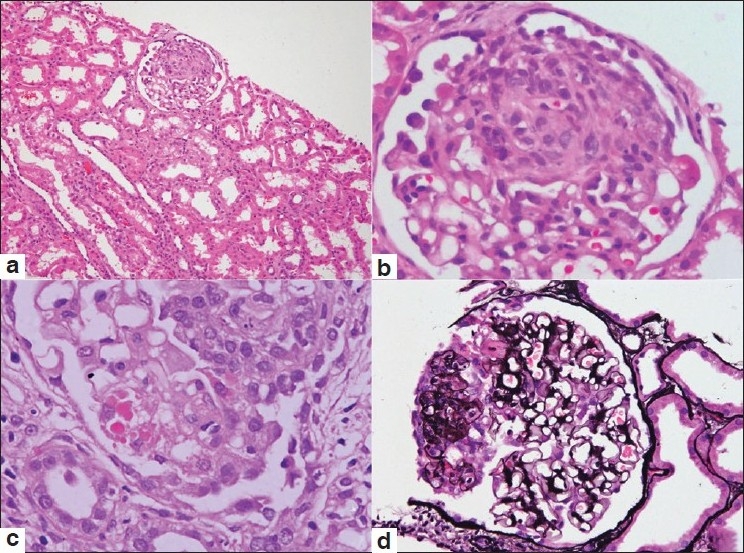
Photomicrographs from cases of collapsing glomerulopathy showing a glomerulus with the collapse of the tuft and hyperplasia of overlying visceral epithelial cells (a, H and E ×100). Higher magnification shows segmental involvement of the glomerular tuft (b, H and E ×400). The visceral epithelial cells demonstrate prominent intracytoplasmic droplets (c, H and E ×400). The silver methenamine stain highlights the segmental tuft collapse associated with hyperplasia of visceral epithelial cells (d, ×200)

The pathologic findings of our nine cases are listed in Table 2. The number of glomeruli was 10.6±2.9. All the biopsies had one or more (12.5–33.3%) glomeruli showing a segmental collapse of the tuft with swollen hypercellular podocytes overlying the collapsed tuft. The podocytes in these foci showed nuclear enlargement, prominent nucleoli, and PAS-positive intracytoplasmic droplets [Figure 1]. Diffuse interstitial fibrosis, involving more than 50% of the cortical parenchyma and accompanying tubular atrophy, was seen in two cases (22.2%) while the other seven biopsies showed mild to moderate degree of tubulointerstitial changes. Moderate to marked arteriolar hyalinosis was noted in five cases (55.5%). Of the nine biopsies, two showed co-existing acute cellular rejection (Banff grade I and IIb). None of the cases showed viral cytopathic effects or histological features suggestive of CNI toxicity (a striped pattern of tubular atrophy/interstitial fibrosis with/without peripheral nodular arteriolar hyalinosis).

**Table 2:** Pathologic characteristics of nine allograft biopsies with collapsing glomerulopathy

	Patient no.								
	1	2	3	4	5	6	7	8	9
No. of glomeruli	12	9	10	4	6	8	13	6	8
Global sclerosis (%)	7 (58.3)	0	0	1 (25)	0	3 (37.5)	2 (15.4)	0	4 (50)
Segmental collapse (%)	4 (33.3)	1 (11.1)	2 (20)	1 (25)	1 (16.6)	2 (25)	4 (30.7)	1 (16.6)	1 (12.5)
Other glomerular injury	–	FSGS in 2, GBM thick	–	FSGS in 1	–	–	–	Mesangial hypercellularity	–
IF/TA	2	2	2	2	3	3	2	1	2
Acute cellular rejection	–	–	ACR IIb	–	ACR I	–	–	–	–
Arteriolar hyalinosis	Marked	Marked	Mild	Mild	Mild	Marked	Marked	Mild	Marked

IF/TA = Interstitial fibrosis/tubular atrophy; IF = Immunofluorescence; GBM = Glomerular basement membrane; ND = Not done.

Immunofluorescence could be performed in only four cases, of which one showed diffuse granular capillary wall staining for IgA, IgG, IgM, and C3 consistent with immune complex glomerulopathy (the primary disease was not known in this patient). Another biopsy showed strong mesangial deposits of IgA along with IgM and C3 while being negative for IgG and C1q, consistent with IgA nephropathy with CG.

Following a diagnosis of CG, cyclosporine was changed to tacrolimus in one patient while another had a shift from azathioprine to mycophenolate mofetil. No specific alterations in the therapeutic regimen were done in other patients. The follow-up details were available in eight patients at a mean duration of 6 months (3–12 months). At the end of the follow-up period, four patients (44.4%) had graft failure (S. creatinine >5 mg/dl or return to dialysis) while the other four patients had functioning grafts with serum creatinine ranging between 1.5 and 3.3 mg/dl.

## Discussion

CG was initially described as “malignant FSGS” in 1978 due to the clinical presentation of rapidly progressive nephrotic syndrome.[1] In the era of HIV pandemic, CG came to be identified as “HIV-associated nephropathy.” In 1986, Weiss *et al*. described a similar renal lesion in HIV-negative patients and the term “collapsing glomerulopathy” was used to indicate this entity.[11] The concept that CG was related to FSGS was introduced by Detweiler *et al*. and the entity was known as “collapsing FSGS.”[2,12] Many authors now preferr to use “collapsing glomerulopathy” on the basis of histologic, pathogenetic, and clinical differences between CG and FSGS.[3]

Both recurrent and *de novo* CG with features similar to those in native kidney have been scantily described in renal allograft biopsies. Only a few reports and small studies were found in the available literature.[4–9,13] One of the studies reported a frequency of 3.2% CG in allografts,[7] comparable to 3.5% noted in the present study. In the previous reports, recurrent CG in renal allografts have presented with nephrotic syndrome with/without graft dysfunction soon after transplantation while *de novo* CG has been diagnosed as late as 74 months posttransplantation.[5,8,13] The median duration of transplant in the present study was 52 months, with one patient diagnosed with CG 98 months after transplantation. All the patients had graft dysfunction, though only one came with pedal edema.

Due to the relatively recent recognition of this entity, there is no consensus on the appropriate therapeutic regimen for CG. This is especially true for CG occurring in renal allografts, either recurrent or *de novo*. Most of the reported cases had progressive worsening of renal functions with return to dialysis at a variable period after the biopsy diagnosis of CG.[6–8] In the study by Meehan *et al*., all five patients developed graft failure within 24 months after the diagnosis of CG.[7] Another study of seven patients reported return to dialysis in five patients within 3–4 months after the diagnosis. One of these five patients had CG in the native kidney.[8] In the present study, four patients had graft failure while the other four patients had functioning grafts and one patient was lost to follow-up.

The present series of nine patients with CG in renal allograft biopsy reemphasizes the fact that CG, a newly recognized entity, is not an uncommon cause of graft dysfunction and usually presents with detectable and significant proteinuria. Accurate recognition of this distinct clinicopathologic entity and its differentiation from FSGS is essential for the appropriate prognostication of an individual patient. Unlike the previously reported cases, the outcome in our patients was not uniformly unfavorable. Of the eight patients in whom follow-up data were available, four had graft failure. Although it is difficult to categorically state the reason for this, the detection of CG early in the course of disease when renal functions were not compromised severely might have contributed to this favorable outcome. In a study by Stokes *et al*., serum creatinine at diagnosis of CG ranged from 3.4 to 9.4 mg/dl.[8] In contrast, our patients had a mean serum creatinine of 2.4 mg/dl, with only two patients having a value of 3.4 and 5.0 mg/dl at the time of biopsy diagnosis of CG.

In conclusion, CG must be recognized as a cause of graft dysfunction, especially in patients with detectable proteinuria. All such patients should be investigated for known associations like viral infections, drug toxicities, and vascular injury. Since the outcome of allografts with collapsing glomerulopathy may not be favorable, a close follow-up is mandatory. More such studies of collapsing glomerulopathy in renal allografts are required to delineate the prognostic significance of this entity.
